# Oxidative stress, fibrosis, and early afterdepolarization-mediated cardiac arrhythmias

**DOI:** 10.3389/fphys.2013.00019

**Published:** 2013-02-15

**Authors:** Hrayr S. Karagueuzian, Thao P. Nguyen, Zhilin Qu, James N. Weiss

**Affiliations:** ^1^Cardiovascular Research Laboratory, Translational Arrhythmia Research Section, David Geffen School of Medicine at UCLALos Angeles, CA, USA; ^2^Cardiovascular Research Laboratory, Departments of Medicine, Division of Cardiology, David Geffen School of Medicine at UCLALos Angeles, CA, USA

**Keywords:** fibrosis, oxidative stress, ventricular fibrillation, bifurcation analysis, calcium window current, early afterdepolarization

## Abstract

Animal and clinical studies have demonstrated that oxidative stress, a common pathophysiological factor in cardiac disease, reduces repolarization reserve by enhancing the L-type calcium current, the late Na, and the Na-Ca exchanger, promoting early afterdepolarizations (EADs) that can initiate ventricular tachycardia and ventricular fibrillation (VT/VF) in structurally remodeled hearts. Increased ventricular fibrosis plays a key facilitatory role in allowing oxidative-stress induced EADs to manifest as triggered activity and VT/VF, since normal non-fibrotic hearts are resistant to arrhythmias when challenged with similar or higher levels of oxidative stress. The findings imply that antifibrotic therapy, in addition to therapies designed to suppress EAD formation at the cellular level, may be synergistic in reducing the risk of sudden cardiac death.

In a previous review, (Weiss et al., 2010) we discussed the ionic basis of early afterdepolarizations (EADs) and summarized recent theoretical and experimental evidence that the synchronization of chaotic EAD dynamics provides both the trigger (rapid focal activation) and the substrate (dispersion of repolarization) to cause polymorphic ventricular tachycardia (PVT), torsade de pointes (TdP), and ventricular fibrillation (VF) (Asano et al., 1997; Choi et al., 2002; Xie et al., 2007, 2010; Sato et al., 2009, 2010). We emphasized that EADs and triggered activity occur when the activation rate of the outward potassium (K) current is sufficiently delayed in the window voltage range of L-type Ca current (I_Ca−L_) to allow I_Ca−L_ to recover from inactivation and gain sufficient amplitude to evoke a propagated wavefront causing premature ventricular complex (PVC). In other words, the time constants of Ca and K currents have to be in resonance with each other for voltage oscillations underlying EADs to occur (Chang et al., 2012a). The kinetic interdependency between these currents explains the failure of EAD formation with agents that prolong action potential duration (APD) outside the I_CaL_ window current voltage range, preventing I_CaL_ from gaining sufficient amplitude to reverse repolarization (Xie et al., 2009; Weiss et al., 2010; Madhvani et al., 2011). In this paper, we briefly review the early work done on EADs and triggered activity, summarize the state of knowledge on the dynamic and ionic basis of oxidative stress-mediated initiation and termination of EAD-mediated triggered activity, and discuss how myocardial structural remodeling in the form of increased cardiac tissue fibrosis critically promotes EAD-mediated VT/VF both in experimental and simulation studies. Finally, we discuss antifibrotic therapy as a potential strategy to reduce sudden death in high risk patients with heart disease.

[Note: Triggered activity is also caused by delayed afterdepolarizations that arise after full action potential repolarization (Wit and Cranefield, 1976; Cranefield, 1977). In this review we focus exclusively on EADs].

## Brief historical background of EADs

The transition from a normal or slow to suddenly rapid electrical activity in cardiac tissue has the potential to precipitate lethal cardiac arrhythmias. An example is the sudden transition to a rapid repetitive activity by depolarizing afterpotentials coined by Cranefield as “triggered activity” (Aronson and Cranefield, 1974; Cranefield, 1975). Unlike automaticity, triggered activity necessarily requires a prior action potential (AP) to initiate repetitive activity (thus the term “triggered automaticity” is a misnomer). One type of triggered activity arises from EADs that interrupt repolarization and reverse dV/dt from negative to positive. This sort of electrical activity was first observed in nerve cells where periods of rapid spiking, i.e., bursting, are followed by quiescent, silent periods before another bursting episode emerges (Connors and Gutnick, 1990). Based on these observations Cranefield wrote: “*I have suggested that in conformity with the terminology used by neurophysiologist, this sort of afterpotential be called an* ‘*early afterdepolarization*’” (Cranefield, 1975, 1977). The defining feature of EADs is that they arises during either phase 2 (January and Riddle, 1989) or phase 3 (Burashnikov and Antzelevitch, 2003; Maruyama et al., 2011) of the AP before the membrane potential has fully repolarized to the resting level.

**Figure 1:**
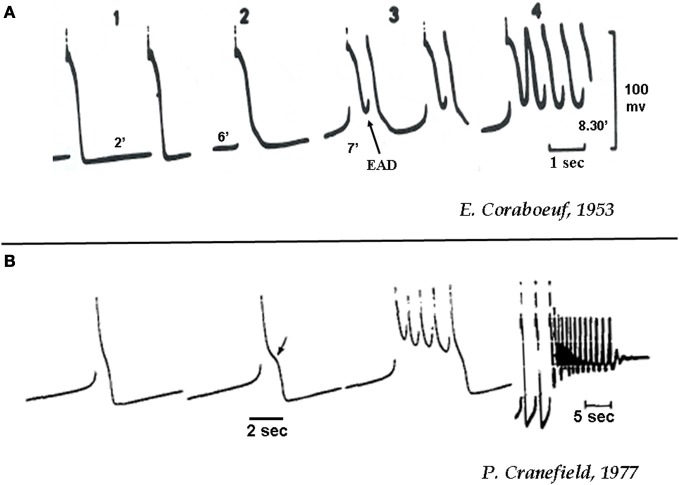
**(A)** Initiation of EAD-mediated triggered activity in canine cardiac Purkinje fibers during acidosis **(A)** and hypokalemia **(B)**. **(A)** The numbers 1–4 indicate progressive increase in the duration (2, 5, 7, 8.5 min) of acidosis (20% CO_2_ and 80% O_2_) as shown below each recording [from Corabeuf, 1953 (Coraboeuf and Boistel, 1953)]. **(B)** Action potentials in a canine cardiac Purkinje fiber exposed to hypokalemia (K^+^_0_ = 2 mmol/L) of increasing duration. Notice that the 3rd beat shows four EAD-mediated action potentials. The 4th recording shows EAD-mediated triggered activity that end with repolarization failure (from Cranefield, 1977).

It is likely that the phenomenon of EAD-mediated triggered activity was already observed by Marcel Segers during the period from 1939 to 1947 using unipolar extracellular recording electrograms in the pre-glass microelectrode recording period (Segers, 1939). His discovery of the association between “*supernormality*” and the “*negative after-potential*” on unipolar electrograms (i.e., depolarizing potential) led him to describe triggered activity this way: “*Extrasystolic arrhythmias could develop under conditions that augment the negative after-potential, such as an increase in the extracellular calcium concentration, aconitine, veratrine, adrenaline, and digitalis*” (Segers, 1942). By using monophasic AP recordings, Segers later showed the presence of “early afterpotentials” and “delayed afterpotentials” which he called *potentiels tardifs* (late potentials). He described the resulting triggered activity as “*battement auto-entretenu du coeur*” (self-sustained beating of the heart) (Segers, 1947). Right after the introduction of glass microelectrode recording technique, Dawes commented in an interesting 1952 review article: “*The recent publication of methods of obtaining membrane action potentials from single cardiac fibres … makes it highly desirable that these observations of Segers should be repeated on such preparations, in order to obtain unequivocal records of these changes*” (Dawes, 1952). The technique of glass microelectrode recording was invented by Ling and Gerard in 1949 to record action potentials from frog skeletal muscle cells (Ling and Gerard, 1949) and in 1951 was adapted by Woodbury and associates for cardiac cell AP recording (Woodbury et al., 1951). Perhaps the first cellular EAD recordings with the glass microelectrode technique was made by Coraboeuf (Coraboeuf and Boistel, 1953) in cardiac Purkinje fibers in 1953. By exposing cardiac Purkinje fibers to elevated partial pressure of carbon dioxide in the perfusion medium to induce acidosis, Coraboeuf demonstrated the EAD-mediated triggered activity shown in Figure 1A (Coraboeuf and Boistel, 1953). In the terminology at the time, he described the observed EADs and triggered activity as “humps,” “second upstroke,” and “reexcitation,” or “repetitive activity” (Coraboeuf and Boistel, 1953). In the ensuing years this potentially arrhythmogenic mechanism was characterized using glass microelectrodes by multiple laboratories, induced by diverse forms of stresses designed to mimic various diseased cardiac conditions. The EAD-inducing stresses included hypoxia by Trautwein et al. (1954) elevated catecholamines by Hoffman and Cranefield (1960) aconitine by Schmidt (1960) extracellular ion manipulation by Hutter and Noble (1961) and reduction of delayed rectifier potassium current (“repolarization reserve reduction”) by Hauswirth et al. (1969). The importance of reduced repolarization reserve in promoting EAD-mediated triggered activity was later directly tested by Katzung who injected a small and steady constant depolarizing currents in isolated papillary muscle which elicited rapid repetitive activity that he called “regenerative discharge of action potentials” or “depolarization-induced automaticity” (Katzung, 1975). The effect of premature stimulation on cesium-induced EADs were investigated by Damiano et al. (Damiano and Rosen, 1984) and the influence of I_Ca−L_ agonists on EADs was assessed by January et al. in short segments of cardiac Purkinje fibers using voltage-clamp technique (January et al., 1988). These studies suggested that EADs are more prone for termination by a premature stimulus at more negative membrane potentials (Damiano and Rosen, 1984) and the EAD arise as a result of reactivation of I_Ca−L_ (January et al., 1988). More recently, reactive oxygen species (ROS) produced either with hydrogen peroxide (H_2_O_2_) (Ward and Giles, 1997; Song et al., 2006; Xie et al., 2009; Madhvani et al., 2011; Nguyen et al., 2012) or angiotensin II (ATII) via the activation of the NADPH oxidase, (Xie et al., 2009; Bapat et al., 2012) and mitogen-activated kinases (JNK, p38) (Bapat et al., 2012) have been shown to potently induce EADs in isolated cardiac myocytes.

## Dynamic and ionic bases of EAD-mediated transient triggered activity

Non-linear dynamics has provided important insights into the mechanisms of cardiac rhythm disorders. Bifurcation theory has been particularly useful in defining and predicting the emergence of cardiac instabilities such as electrical alternans and EAD-mediated triggered activity. A bifurcation occurs when an alteration in the value of one or more parameters in a system causes a qualitatively new behavior to emerge. For example, the phenomenon of APD alternans, caused by steep slope APD restitution curve, (Nolasco and Dahlen, 1968) is an example of bifurcation whereby a critical rise in the pacing rate (the bifurcation parameter) suddenly causes APD to alternate between long and short values. The dynamic bifurcation sequence also explains very complex and irregular cellular dynamics (chaos) that arise at still faster rates of pacing (Guevara et al., 1981, 1984; Chialvo and Jalife, 1987; Chialvo et al., 1990; Karagueuzian et al., 1993). The slope of the APD restitution curve (a plot of APD vs. previous diastolic interval) regulates APD alternans under diverse cardiac conditions, (Garfinkel et al., 2000) and its magnitude has been used as a marker for electrical instability causing dispersion of repolarization, wavebreak and VT/VF (Karagueuzian et al., 1993; Garfinkel et al., 2000; Weiss et al., 2006). Later studies further refined the APD restitution hypothesis by incorporating the independent contribution made by the intracellular Ca ([Ca^+2^_i_]) dynamics and conduction velocity (CV) restitution in promoting APD alternans (Weiss et al., 2006).

Recent dynamical analysis has revealed a bifurcation mechanism underlying EAD-mediated triggered activity in cardiac tissue, namely dual Hopf-homoclinic bifurcations (Tran et al., 2009; Chang et al., 2012a). In this scenario, the voltage oscillations underlying EADs are generated by a Hopf bifurcation, and are eventually terminated by a homoclinic bifurcation. A Hopf bifurcation is a dynamical process by which an equilibrium (in this case, the plateau voltage) becomes unstable and begins to oscillate, and is a common bifurcation to many physical and biological phenomena. For example, the collapse of the Tacoma Narrows Bridge in 1940 was attributed to a Hopf bifurcation. Initially the cause of the collapse was attributed to an external periodic (oscillatory) force produced by the wind. However, later analyses suggested that the mechanism and the origin of the oscillations were actually the result of the emergence of an unstable combination of air speed and torsional stiffness of the bridge triggering self-induced oscillations (via Hopf bifurcation) that eventually led to the collapse of the bridge (Billah and Scanlan, 1991). In biological systems, Hopf bifurcations have been proposed to underlie biological oscillation such as cell cycle, (Qu et al., 2003) pacemaking in neurons and heart, (Guevara and Jongsma, 1992) glycolytic oscillations, (Goldbeter and Lefever, 1972), and circadian rhythms (Goldbeter, 1995).

In the case of EADs, the requirements for voltage to oscillate during phase 2 or phase 3 of the cardiac AP depend strongly on the properties of I_Ca−L_ in relation to repolarizing outward currents. Oscillatory behavior emerges in the I_Ca, L_ window voltage range (−40 to 0 mV), where the steady state I_Ca−L_ activation and inactivation curves overlap, but can occur only when the activation of time-dependent outward currents, particularly I_Ks_, is delayed as in the setting of reduced repolarization reserve (Tran et al., 2009; Madhvani et al., 2011; Chang et al., 2012a). The presence of Ca waves may act synergistically with I_Ca, L_ reactivation to promotes EADs (Volders et al., 2000; Zhao et al., 2012). The Hopf bifurcation initiates the membrane oscillations, causing single or multiple EADs. As the outward currents slowly activate, the system gradually approaches and passes the homoclinic bifurcation, at which point the oscillations (EADs) are destroyed and the AP fully repolarizes. The Hopf-homoclinic bifurcation hypothesis of EAD formation was rigorously validated using a three variable ventricular action potential cell model in which the rate constants of activation and inactivation of the L-type Ca current (I_Ca−L_) and the rate constant of activation of the delayed rectifier K current (I_K_) were varied. The Hopf-homoclinic bifurcation sequence required that the time constants of these currents were properly matched (Tran et al., 2009; Chang et al., 2012a). Briefly, EADs were produced when the activation rate of I_K_ was slow enough to allow the AP plateau voltage to drift gradually into the I_Ca−L_ window voltage range. In addition, the voltage dependence of the I_Ca−L_ steady-state activation and the inactivation curves had to be steeply sloped (Tran et al., 2009; Sato et al., 2010; Chang et al., 2012a). This scenario was reproduced experimentally in isolated rabbit ventricular myocytes using a hybrid biological–computational approach (dynamic clamp) to introduce a virtual I_Ca−L_ with programmable properties into a patch-clamped ventricular myocyte, such that the biophysical properties of the virtual I_Ca−L_ could be manipulated at will (Madhvani et al., 2011). More recently, we extended the computational analysis to a more realistic cardiac cell model incorporating detailed Ca cycling and ionic fluxes (Chang et al., 2012a). Under these conditions, we found that long bursts of EAD-mediated triggered activity could be induced, whose interspike frequency gradually slowed before the burst terminated. The latter frequency-slowing prior to burst termination feature is a classic dynamical signature of the Hopf-homoclinic bifurcation mechanism, previously noted in bursting neurons, (Song and Xu, 2009) and agreed with experimental observations in cultured neonatal rat ventricular myocyte monolayers. In this case, EAD burst termination was mediated by progressive intracellular Na accumulation during the burst, which gradually increased repolarization reserve by stimulating electrogenic Na-K pump outward current (I_NaK_) (Chang et al., 2012a). We confirmed this ionic mechanism of termination of the triggered activity by clamping the intracellular Na in the cell model, which caused the bursting to continue indefinitely.

An important prediction of the dual Hopf-homoclinic bifurcation mechanism is that EADs occur only over a critical range of reduced repolarization reserve, and can be terminated by either increasing or further decreasing repolarization reserve (Tran et al., 2009; Chang et al., 2012a). The latter prediction was experimentally tested in cardiac monolayers treated with BayK8644 and isoproterenol to induce bursts of EAD-mediated triggered activity. Further reducing repolarization reserve by blocking the transient outward K current with 4-amino pyridine, or by blocking the Ca^2+^-CaM-dependent inactivation of the I_Ca−L_ by overexpressing mutant Ca^2+^-insensitive calmodulin (CaM_1234_) further prolonged APD but suppressed EAD-mediated triggered activity (Chang et al., 2012a). These findings emphasize the importance of the membrane voltage gradually entering the I_Ca−L_ window voltage range for EADs to occur. If the membrane voltage during the AP plateau remains above 0 mV, EADs are unlikely to occur, regardless of how long the APD is. This finding accounts for previous observations that AP triangulation (where the AP plateau gradually drifts below 0 mV during repolarization) is much more arrhythmogenic than APD prolongation in which the AP maintains a high plateau voltage (Shah and Hondeghem, 2005; Hondeghem, 2008). Finally, when repolarization reserve is reduced even further, repolarization failure occurs and the membrane potential remains depolarized. Under these conditions, small amplitude membrane oscillations can result from intracellular Ca waves, which activate Ca-sensitive currents, such as in isolated cardiac myocytes exposed to oxidative stress with H_2_O_2_ and ATII and hypokalemia (Figure 1B) (Cranefield, 1977; Bapat et al., 2012; Nguyen et al., 2012). Dynamically, repolarization failure arises from a different type of bifurcation, called saddle-node bifurcation which results in bistability (Chang et al., 2012b).

## Oxidative stress, repolarization reserve reduction, and EADs

Mammalian cells utilize redox signaling via ROS such as H_2_O_2_ to regulate diverse physiological pathways including cell proliferation, differentiation, and migration (Sundaresan et al., 1995; Rhee, 2006). Due to the potential toxicity when ROS levels rise excessively, H_2_O_2_ has been described as “*a necessary evil for cell signaling*” (Rhee, 2006). Thus, the concept of redox signaling was recognized not only by its regulatory role in normal physiological processes but also by its influence on disease progression, (Cave et al., 2006; Rhee, 2006) including its demonstrated ability to enhance cardiac vulnerability to arrhythmias in animal and human studies (Corretti et al., 1991; Carnes et al., 2001; Lokuta et al., 2005; Erickson et al., 2008; Morita et al., 2009; Erickson et al., 2011). Oxidative stress reduces repolarization reserve by enhancing late I_Na_ (I_Na−L_), I_Ca−L_, and the Na-Ca exchanger to favor the formation of EADs (Corretti et al., 1991; Ward and Giles, 1997; Song et al., 2006; Xie et al., 2009; Zhao et al., 2011). Oxidative stress also inhibits glycolysis, (Corretti et al., 1991) a major supplier of ATP to the sarcoplasmic-endoplasmic reticulum Ca^2+^-activated ATPase (SERCA) pump (Xu et al., 1995; Morita et al., 2011a) that reduces the SR Ca uptake further adding to dysregulation of intracellular Ca cycling dynamics. Finally, while other enzymatic pathways may also be activated by oxidative stress such as xanthine oxidase causing afterpotentials like hydrogen peroxide, (Goldhaber, 1996) the purpose of this review however, is to exclusively focus on the enzymatic pathways shown to induce afterpotentials and triggered activity causing VT/VF.

Recently, a major pathway by which oxidative stress has been shown to increase I_Ca−L_ and late I_Na−L_ is via activation of Ca-calmodulin dependent protein kinase II (CaMKII) (Ward and Giles, 1997; Song et al., 2006; Erickson et al., 2008; Xie et al., 2009; Zhao et al., 2011). H_2_O_2_-mediated activation of CaMKII occurs by the oxidation of paired methionine residues (M281/282) in the regulatory domain of CaMKII which removes the auto-inhibition of the enzyme by freeing the catalytic site causing sustained CaMKII activity even in the absence of Ca^2+^/CaM (Erickson et al., 2008). CaMKII oxidation could be reversed by methionine sulfoxide reductase A (MsrA) and MsrA knockout mice show exaggerated CaMKII oxidation causing myocardial apoptosis, impaired cardiac function, and an increased mortality after myocardial infarction (Erickson et al., 2008). Oxidative stress is directly induced by ATII via the nicotinamide adenine dinucleotide phosphate (NADPH) oxidase system, which, like exogenous H_2_O_2_, leads to oxidative activation of CaMKII (Zhao et al., 2011). Indeed, ATII (1–2 μM) increases ROS fluorescence in cardiac myocytes to promote EADs that could be abolished by NADPH oxidase inhibitor, apocynin (Zhao et al., 2011). As in the case of the H_2_O_2_, oxidative stress caused by ATII activates I_Ca−L_ and I_Na−L_ causing reduced repolarization reserve and promotion of EADs (Zhao et al., 2011). These results indicate that CaMKII can integrate Ca^2+^ and ROS signaling to promote EADs in isolated myocytes (Erickson et al., 2008; Xie et al., 2009; Zhao et al., 2011).

### EADs at the isolated myocyte vs. intact tissue level

**Figure 2:**
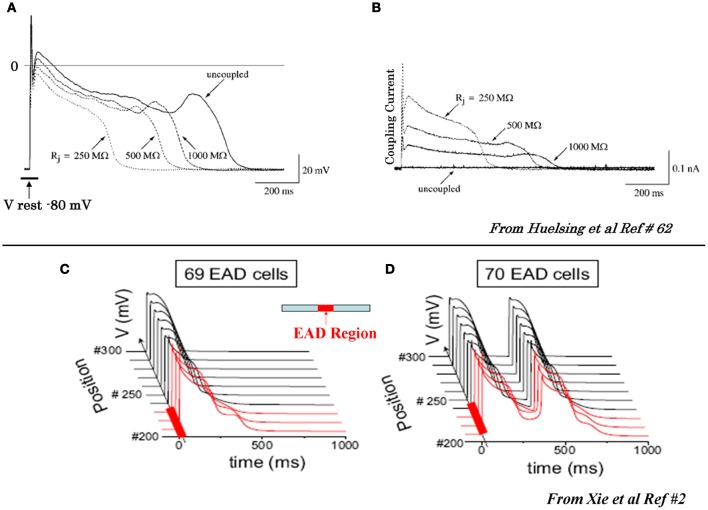
**Suppression of EAD by coupling to a cell with no EAD (A) and (B) and initiation of propagating EADs in 1D cable with a critical number of cell synchronously firing EADS (C) and (D). (A)** In the uncoupled state (solid traces) the EAD is apparent, however, as the coupling resistance between the EAD cell and the non-EAD cell with a resting potential of −80 mV is progressively reduced the amplitude of the EAD decreases and eventually it is suppressed at coupling resistances >500 MW. **(B)** Intercellular current flow during coupling of the two cells with decreasing resistances (dashed traces) shown in panel **(A)**. The solid trace shows zero current flow between the two cells in the uncoupled state (from Huelsing et al., 2000). Panels **(C)** and **(D)** show AP traces along a 1D cable, with the red traces indicating the EAD-susceptible cells in the central region (middle diagram), and the normal non-susceptible cells in black. The EAD failed to propagate with 69 susceptible cells in the central region **(C)**, but propagated successfully with 70 susceptible cells **(D)** (from Xie et al., 2010).

While oxidative stress caused by H_2_O_2_ and ATII readily initiate EADs in isolated cardiac myocytes, (Ward and Giles, 1997; Song et al., 2006; Xie et al., 2009; Zhao et al., 2011; Bapat et al., 2012; Nguyen et al., 2012) an obvious question arises if these same stressors also initiate EAD-mediated triggered activity in intact hearts. We investigated this question in intact normal adult rat and rabbit hearts using isolated-perfused Langendorff preparations. We found that both H_2_O_2_ and ATII, which readily promoted EADs in single isolated myocytes, consistently failed to promote EADs in intact hearts from normal young adult rats and rabbits even when we raised the level of these two stressors beyond the level that promoted EADs in the isolated myocytes (Morita et al., 2009, 2011a,b; Sato et al., 2009; Bapat et al., 2012). Since EADs typically occur irregularly, rather than consistently from beat-to-beat, the discrepancy between isolated myocytes vs. intact normal heart may result from the powerful source-sink relationship in well-coupled cardiac tissue. That is, if a few EAD-generating cells (i.e., “source”) are well-coupled to many normally repolarizing cells with no overt EADs (i.e., “sink”), the source-sink mismatch may prevent the EADs from manifesting in tissue (Xie et al., 2010). The importance of diffusive electrotonic cellular coupling on the modulation of EADs is demonstrated by coupling two isolated myocytes, one with EADs and the other without EAD, via a variable resistor. With infinite resistance between the two cells (i.e., complete cellular uncoupling), the EADs readily appear in the cell that produces EAD. However, when electrical coupling is applied between the two cells, the amplitude of the EAD producing cell starts to lose amplitude as the intercellular coupling conductance between the two cells increases and with a sufficiently high coupling conductance the EAD-producing cell no longer produces EADs. Figure 2 illustrates the phenomenon of EAD suppression in a cardiac myocyte by electrotonic coupling (Saiz et al., 1999; Huelsing et al., 2000). Our simulation studies using a realistic cardiac cell model in Three dimensional (3D) normally-coupled tissue showed that one needs ≈700,000 contiguous cells that synchronously produce EADs in order to overcome the source-sink mismatch and allow a triggered PVC to propagate (Xie et al., 2010). In two dimensional (2D) tissue this number decreases to ≈7000, and in a 1D cable to ≈70. Interestingly and as expected, the minimum number of cells needed to produce a propagated EAD-induced PVC in 3D tissue decreased by as large as 40 fold when cell-to-cell gap junctional coupling is reduced (Xie et al., 2010). Fibrosis, which imposes insulating collagen bands between strands of myocytes (essentially converting 3D tissue into a network of 1D cables), was even more potent at reducing the number of myocytes required for PVC formation. Figure 2 illustrates our methods of assessing the importance of cellular coupling in the genesis of EAD in one dimensional (1D) cable (Xie et al., 2010). These experimental and simulation studies strongly suggest that the observed discrepancy in EAD production between isolated myocytes and normally-coupled cardiac tissue may result from the source-sink mismatch. In a well-coupled cardiac tissue, the source-sink mismatch provides a powerful protective effect to suppress EAD formation by the delinquent cells, preventing the emergence of cardiac arrhythmias. Based on these findings, it could be surmised that cardiac diseases that cause myocyte decoupling should facilitate the emergence EADs when the repolarization reserve is reduced. A number of factors reduce cellular coupling in diseased heart, including gap junction remodeling and fibrosis. Fibrosis forms insulating barriers between cells and group of cells by interstitial tissue deposits of collagenous filaments by the proliferating cardiac myofibroblasts (Weber and Brilla, 1991; Wynn, 2007). Aging in rats and rabbits is known to manifest not only increased cardiac fibrosis but also down-regulation of the gap junctional connexins43 (Cx43) (Sato et al., 2009; Morita et al., 2011a) that effectively reduce the coupling conductance between cardiac myocytes.

### Role of fibrosis

Traditionally, increased arrhythmic risk due to increased cardiac fibrosis has been attributed to alteration in conduction and reentry. The early seminal studies by Spach and associates have shown that fibrotic changes in cardiac tissue that emerge in aged hearts, reduces the safety margin for conduction by promoting conduction block and reentry when the availability of the fast sodium current (“source”) was reduced by premature electrical stimulation (Spach et al., 1989, 2007).

**Figure 3:**
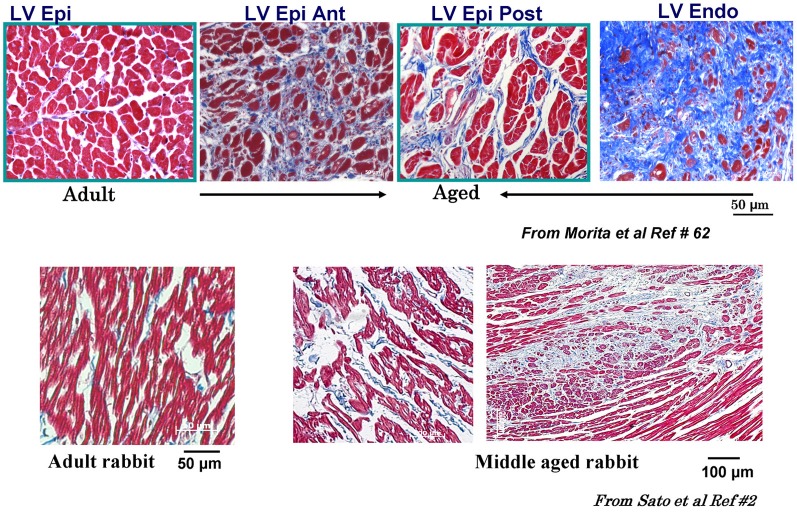
**Masson trichrome staining in adult and aged rat and middle-aged rabbit ventricles.** Notice increased fibrosis in the aged compared to adult LV (top figures) with almost complete fibrosis at the endocardium (Endo) with intermediate fibrosis of the posterior left ventricle (LV) and the septum (blue color). Lower panels show LV fibrosis in the middle aged compared to adult rabbit LV. Ant, anterior; RV, right ventricle; Post, posterior; Epi, epicardium [from Sato et al., 2009 and Morita et al., 2009 (Lokuta et al., 2005)].

**Figure 4:**
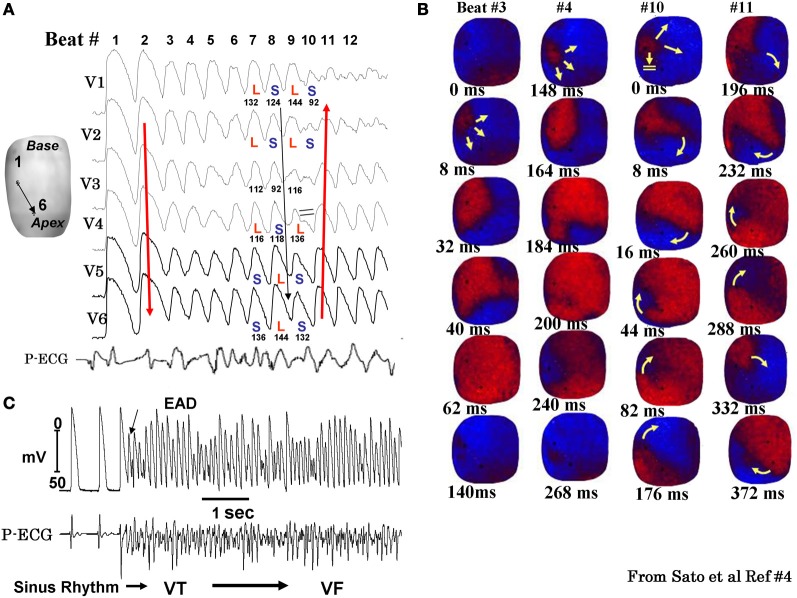
**Spontaneous initiation of EAD-mediated VF in a middle-aged rabbit exposed to 0.1 mM H_2_O_2_.** Panel **(A)** shows 6 simultaneously epicardial optical APs (V1 to V6) recorded from the sites shown on the left silhouette of the heart. After nine EAD-mediated focal activations arising from the base of the heart (panel **B**) the wavefront undergoes block at mid LV anterior wall (beat #10) after the emergence of a spatially discordant APD alternans shown in panel **(A)** (L and S stand for short APD respectively). The wavefront however, continues to propagate lateral to the site of block in a clockwise direction causing the formation of a reentrant wave of excitation as shown in the snap shots of beat #11 in panel **(B)**. The red color in the snap shots represents depolarization and the blue repolarization. The yellow arrows in the snap shots represent the direction of the wavefront propagation with double horizontal lines denoting the site of conduction block. The numbers under each snap shot is activation time starting with arbitrary zero ms for beast #3 and #4 and then again for beats #10 and #11. Panel **(C)** is a microelectrode recording of another VF episode recorded in the same heart showing spontaneous initiation of VF by a mechanism compatible with EAD-mediated TA (from Sato et al., 2009).

While the alterations of cardiac conduction and the associated reentrant arrhythmias in a fibrotic cardiac tissue are well-recognized both in animal (Spach, 1991; Hayashi et al., 2002, 2003) and human studies, (de Bakker et al., 1988; Wu et al., 1998) the influence of cardiac tissue fibrosis on the genesis of EADs during oxidative stress remained poorly explored. In view of the discrepancy between the ability of oxidative stress to induce EADs in isolated myocytes, but not in intact hearts from young rats or rabbits, we hypothesized that cardiac tissue fibrosis would facilitate the generation of EADs and triggered activity in intact hearts exposed to oxidative stress by altering source-sink relationships. Since aged hearts exhibit increased fibrosis, we evaluated the influence of H_2_O_2_ and ATII to promote EAD-mediated arrhythmias in isolated Langendorff-perfused hearts from aged rat (~2 years old) and middle aged rabbit (~5–6 years old) hearts, using high resolution optical mapping for cardiac activation pattern and glass microelectrode cellular recordings from selected left ventricular (LV) epicardial sites. Aging-related fibrosis is apparent in these two species and manifested high degree of heterogeneity in its distribution being very dense in the endocardium with intermediate levels fibrosis on the anterior and posterior LV wall as shown in Figure 3. In sharp contrast to adult normal hearts, the aged hearts of both species when exposed to oxidative stress with H_2_O_2_ or ATII readily promoted EADs and triggered activity that led to VT and then to VF (Morita et al., 2009, 2011b; Bapat et al., 2012). Figure 4 illustrates an episode of EAD-mediated triggered activity in a middle-aged rabbit heart causing a focal VT which then degenerates to VF. The focal VT caused by EAD-mediated triggered activity originated from the base of the heart, which then underwent functional conduction block due to the emergence of spatially discordant APD alternans. Similarly, in the aged fibrotic rat hearts oxidative stress with H_2_O_2_ promoted EADs leading to triggered activity, VT and VF. In addition to fibrosis, both species showed down-regulation of total Cx43 as indicated by reduced distribution of detectable Cx43 immunostaining positive spots in the LV tissue (Morita et al., 2011b).

**Figure 5:**
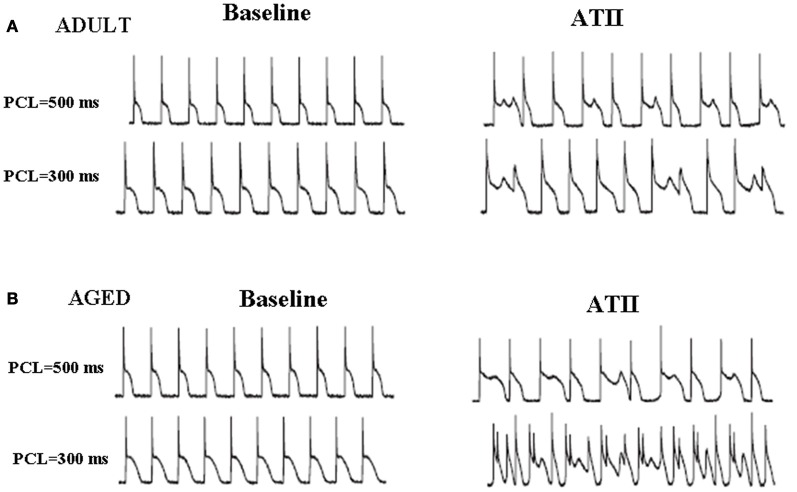
**Angiotensin II induces EADs both in young/adult (A) and aged (B) isolated LV myocytes.** Effects of ATII (2 μmol/L) in both age groups are shown at two different pacing cycle lengths (PCLs). Both young and aged myocytes manifested EADs and triggered activity with significant differences between the two aged groups (from Bapat et al., 2012).

It could be argued that the observed differences between the two age groups may result from aging-related ventricular myocyte electrical remodeling whereby the sensitivity of the aged cardiomyocyte to oxidative stress greatly increases relative to young/adult cardiomyocytes (Anyukhovsky et al., 2002; Sudhir et al., 2011). To investigate this possibility, we directly compared the susceptibility of isolated single rat ventricular myocytes isolated by enzymatic dissociation from young and aged rats and subjected them to oxidative stress and studied them under patch-clamp conditions. We exposed isolated ventricular myocytes from both age groups to three different clinically-relevant stressors, namely ATII, (Zhao et al., 2011) hypokalemia, (Osadchii, 2010), and H_2_O_2_ (Nguyen et al., 2012). Our results show that both young and aged isolated myocytes responded similarly to these three stressors and readily induced EADs and triggered activity. These findings suggest that electrical remodeling is not a major cause for the observed age-dependent differences to stress-induced EADs, triggered activity and VT/VF at the whole heart level. Figure 5 illustrates an example.

### Potential arrhythmogenic influences of myocyte-myofibroblast coupling

Normal adult hearts contain more non-myocytes than myocytes, with fibroblasts making up the majority of non-myocytes (Wynn, 2007; Kohl and Camelliti, 2012). Fibroblasts become activated by injury and stress as part of a normal wound-healing response, and differentiate into myofibroblasts which proliferate and secrete collagen, smooth muscle α-actin, and stretch-sensitive ion channels, and connexins (Camelliti et al., 2004). Co-culture studies have shown that heterocellular coupling readily develops between myocytes and myofibroblast via gap junction Cx43 molecules (Gaudesius et al., 2003; Miragoli et al., 2007; Jacquemet and Henriquez, 2008; Vasquez et al., 2011). Since myofibroblasts have a less negative resting membrane potential, they can depolarize myocytes sufficiently to induce spontaneous pacemaker activity when the myofibroblast population exceeds 15% in co-cultures (Miragoli et al., 2007). While such heterocellular couplings seen in tissue co-culture may play an active role in arrhythmogenesis, at present definitive evidence that fibroblasts or myofibroblasts form gap junctions with myocytes in intact native heart tissues remains controversial. While awaiting a final verdict to this important question, investigations as to how myocyte to myofibroblast (M-MF) coupling may alter myocyte electrophysiology remain of considerable interest in cardiac arrhythmogenesis.

**Figure 6:**
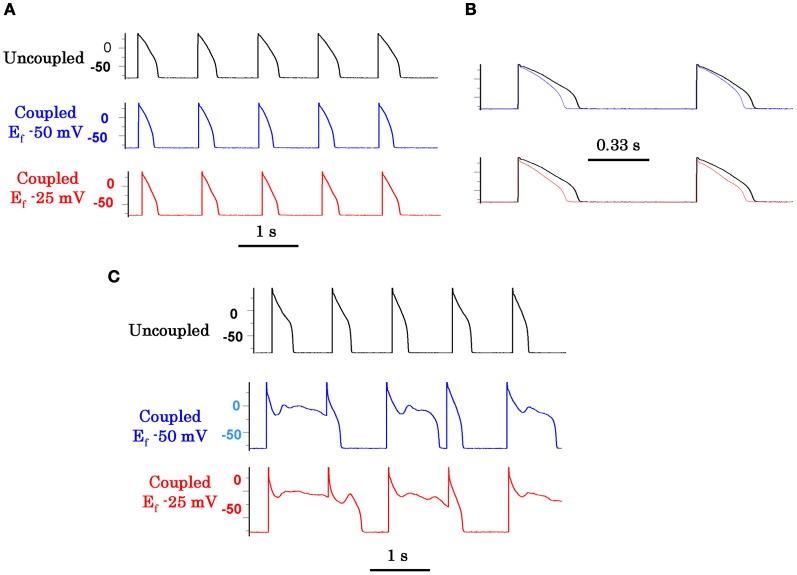
**Promotion of EADs by myofibroblast-myocyte coupling (M-F) during oxidative stress.** Coupling a patch-clamped myocyte superfused with normal Tyrode's solution to a virtual fibroblast promotes no EADs **(A)**. However, coupling lowers the AP plateau and shortens APD as shown in the superimposed traces at a faster time scale in panel **(B)**. Panel **(C)** (top traces) shows lack of EAD induction with 0.1 mmol/L H2O2 in the uncoupled state. However, coupling the myocyte to a virtual fibroblast (C_f_ 6.3 pF, Gj 3.0 nS) caused EADs to reappear, which were more prominent when Ef was −25 mV (bottom traces) than −50 mV (middle traces). C_f_ is fibroblast capacitance in pF, Gj is M-F gap junctional conductance in nS, and E_f_ is the resting potential of the fibroblast in mV (from Nguyen et al., 2012).

We investigated the influence of M-MF coupling on the genesis of EADs under conditions of reduced repolarization reserve using the dynamic clamp technique to electronically couple an isolated ventricular myocytes to a virtual “myofibroblast” endowed with physiologically realistic membrane capacitance, resistance, voltage and gap junctional coupling resistance (MacCannell et al., 2007). With this technique we sought to systematically define the characteristics and the mechanisms by which the coupled fibroblasts influence susceptibility to EADs. We found that M-MF coupling promotes an I_to_-like outward current in the cardiac myocyte (Nguyen et al., 2012). The I_to_-like current reflects the charge movement required to depolarize the fibroblast membrane capacitance from the coupled resting potential to the AP plateau. This I_to_-like current promotes EADs by lowering the AP plateau voltage into the I_Ca−L_ window current range (Nguyen et al., 2012). Not only does this allow I_Ca−L_ to reactivate, but it also slows the activation of I_Ks_ by depressing the plateau voltage. Figure 6 illustrates this facilitative role of M-MF coupling in the induction of EADs. The importance of the I_to_-like current in the genesis of EAD is clearly demonstrated by the suppression of the EADs when the I_to_-like component was eliminated by the dynamic clamp protocol (Nguyen et al., 2012; Workman et al., 2012). It should be noted that I_to_ can also suppress EADs when its amplitude or pedestal current becomes sufficiently large to overcome I_Ca−L_ reactivation (Workman et al., 2012).

### M-MF coupling and EAD propagation in 2D simulated cardiac tissue

**Figure 7:**
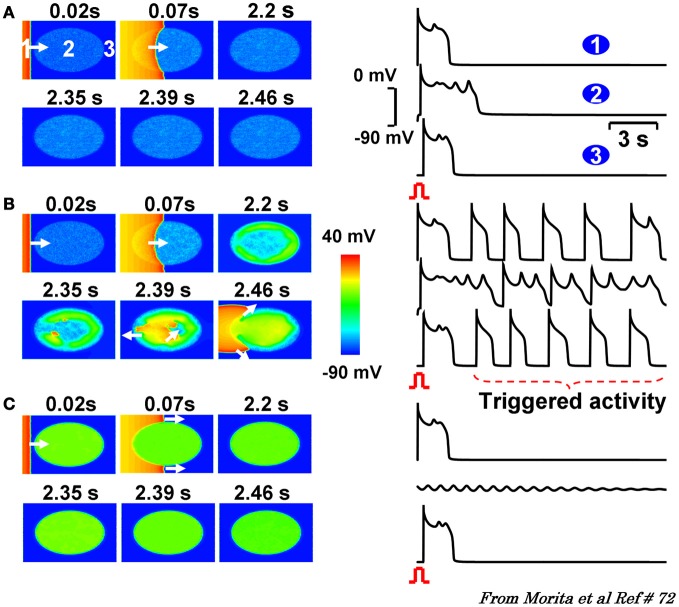
**2D simulations in a tissue with progressive regional increase in the number of fibroblasts to myocyte gap junction couplings.** Panels **(A–C)** are voltage snap shots after a paced beat from the left border of the tissue (red) with central ellipse (site 2) representing the region of progressive increase in fibroblast to myocyte (F-M) coupling ratio. In panel **(A)** with F-M ratio of 1, promotes small amplitude non-propagating EADs. Similar dynamic scenario is also observed when the F-M ratio in the ellipse is raised to 3 as shown in panel **(C)**. After the paced beat, EADs die out locally under these two conditions of M-F coupling. However, when the F-M ratio is in the intermediate range (1.1, panel **B**) the EADs and triggered beats are now of higher amplitude that propagate and excite the adjacent cardiac tissue as triggered beats. The numbers on top of each snap show denote time in seconds after the onset of paced beat from the left of the tissue. White arrows indicate the direction of wavefront propagation [From Morita et al., 2011a,b (Goldhaber, 1996)].

Given the extreme heterogeneity of myocardial tissue fibrosis, we investigated the importance of the level of fibrosis on initiation and propagation of EADs. The level of fibrosis in our model was graded by the number of fibroblasts that couple to a single cardiomyocyte under conditions of reduced repolarization reserve so to mimic oxidative stress. We found a critical level of fibrosis (i.e., number of myofibroblast coupled to myocytes) was needed to promote propagated EADs. That is either too few or too many number of coupled fibroblasts prevented the genesis (too few) or propagation (too many) of the EADs. A “critical” level of fibrosis was needed for both initiation and the propagation of the EAD to cause arrhythmias (Morita et al., 2009). Figure 7 illustrates this phenomenon.

## EADs, fibrosis, and VT/VF in humans

Perhaps the first experimental study linking persistent I_Na−L_ to EADs was made with the use of sea *Anemonia sulcata* toxin II (ATX II) (Isenberg and Ravens, 1984). This early experimental observation did not remain an isolated laboratory curiosity as it was later discovered that a congenital form of a long QT syndrome (LQTS) in humans, LQT3, is associated with a persistent I_Na−L_ causing APD prolongation (Bennett et al., 1995) and a propensity for TdP, VT, and VF (Moreno and Clancy, 2012). The presence of I_N−La_ during the plateau phase of the AP can critically reduce repolarization reserve despite its small magnitude (range 20–60 pA), and coincides in time with the reactivation kinetics of I_Ca−L_(Madhvani et al., 2011) to promote EADs (Xie et al., 2009). It is suggested that EADs caused by human cardiomyocytes lead to triggered activity causing PVT and TdP, (Ruan et al., 2009) the primary arrhythmia mechanism and cause of sudden cardiac death in LQT3 carriers (Clancy and Kass, 2005). In addition to congenital LQT3, ventricular myocytes isolated from human end-stage failing hearts manifest EADs during adrenergic stimulation, unlike non-failing myocytes (Veldkamp et al., 2001).

**Figure 8:**
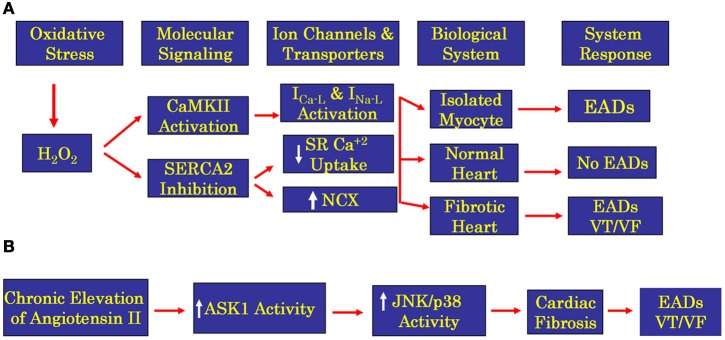
**Flow chart showing oxidative stress signaling pathways for H_2_O_2_ (panel A) and ATII (panel B) in isolated ventricular myocytes and whole normal and fibrotic hearts.** While EADs are readily induced by the proposed oxidative signaling pathways in the isolated ventricular myocytes, the initiation of EADs and VT/VF in the whole heart is restricted only to structurally remodeled heart with increased fibrosis. Induction of fibrosis by either H_2_O_2_ or ATII promotes EADs in isolated cardiac myocytes however, at the whole heart level only the presence of enhanced cardiac fibrosis could lead to propagated EADs by the mechanism of triggered activity leading to VT/VF. ASK1 is apoptosis signaling kinase 1, is a member of mitogen-activated protein kinase kinase kinase family that activates JNK (c-Jun N-terminal kinase) and p38 kinase (p38 mitogen-activated kinase) eventually initiating transcription causing hypertrophy and fibrosis.

While cardiac diseased conditions are often associated with pro-oxidant state and reduced repolarization reserve that could promote EAD-mediated VT/VF, recent clinical studies provide mounting evidence that increased cardiac fibrosis may also be an independent predictor of VT/VF in humans (Klem et al., 2012; Leyva et al., 2012). In our animal models of EAD-mediated VT/VF, a simultaneous reduction in repolarization reserve and a critical increase in cardiac fibrosis are necessary to promote VT/VF. Evidence is mounting that human hearts also require a similar “two-hit” scenario (reduced repolarization reserve and fibrosis) to initiate VT/VF. Quantitative measurement of cardiac fibrosis inhuman poses a great challenge. Early studies relied on myocardial biopsy samples to assess the presence and quantify myocardial fibrosis, clearly an inadequate approach to assess global cardiac fibrosis. Since the demonstration of the late gadolinium enhancement in cardiovascular magnetic resonance imaging in patients with non-ischemic dilated cardiomyopathy (DCM) to be similar to the cardiac fibrosis determined by histological examination at autopsy, (McCrohon et al., 2003) attempts have been made to quantify global fibrosis in patients with DCM using enhancement as a surrogate of fibrosis. The results of these early studies showed that the extent of myocardial fibrosis was an independent predictor for VT/VF in patients with DCM (O'Hanlon et al., 2010) and a critical level of fibrosis was found necessary to be predictive of sudden cardiac death and the number of ICD discharges in these patients (Klem et al., 2012; Leyva et al., 2012). The fibrosis predictive ability of the occurrences of sudden cardiac death and ICD discharges reaches a plateau at scar sizes between 5% and 20% of the LV volume, with larger fibrosis sizes tending to decrease the incidence of sudden cardiac death and ICD discharges (Klem et al., 2012). This observation is compatible with our experimental (Morita et al., 2009) and 2D simulation studies where we show the need for a critical level of fibrosis to promote propagated EADs (Figure 7). These early studies suggest that fibrosis could be used as a marker for risk stratification of sudden cardiac death, (Sovari and Karagueuzian, 2011; Klem et al., 2012; Leyva et al., 2012) and that imaging cardiac fibrosis with enhancement appears to more accurately reflect the presence and extent of cardiac fibrosis than ejection fraction. For example, patients with DCM and fibrosis s may have preserved ejection fraction (Biagini et al., 2012) and conversely others without myocardial scarring and fibrosis manifest severely reduced ejection fraction because of intrinsic depression of cardiac muscle contractility. Figure 8 illustrates schematically our current understanding of the mechanisms involved in oxidative stress-mediated EAD in promoting VT/VF.

### Antifibrotic and antiarrhythmic drug therapy in human VT/VF

Therapy for the management of EAD-mediated VT/VF in fibrotic hearts can be targeted either to critically increase repolarization reserve and/or decrease cardiac fibrosis. At present, selective antiarrhythmic drugs that specifically target the kinetics of activation and inactivation of I_Ca−L_ and outward potassium currents without affecting excitation-contraction coupling are still lacking. It is becoming increasingly evident that targeting the therapy against cardiac fibrosis may be a viable alternative to prevent VT/VF. While data on the efficacy of antifibrotic therapy against VT/VF in humans is sparse, indirect clinical and direct experimental findings suggest the possibility that reduction of myocardial tissue fibrosis reduces the recurrences of VT/VF.

Antifibrotic therapy may be targeted against elevated and disproportionate collagen accumulation or against myofibroblast proliferation or both. The study by Lopez et al. showed that torsemide, a loop diuretic, reduces ventricular collagen volume fraction (CVF) in patients with chronic heart failure (Lopez et al., 2007). Torsemide inhibits the enzyme involved in myocardial extracellular generation of collagen type I molecules (i.e., procollagen type I carboxy-terminal proteinase or PCP) thus reducing myocardial CVF assessed in right septal endocardial biopsies from patients with chronic heart failure (Lopez et al., 2007). Once fibrosis emerges in the heart its speed of expansion is a relatively fast phenomenon and therefore antifibrotic measures need to start sooner rather than later. A recent study in patients with hypertrophic cardiomyopathy (HCM), fibrosis was assessed by gadolinium enhancement MRI at two time points separated by about a 2 years period. It was found that the enhancement increment was a relatively fast event and was directly related to the worsening of the clinical outcome of the patients (Todiere et al., 2012). During a median follow-up of 24 months a sharp step-up of SCD and ICD discharges was observed for scar size >5% of the LV mass (hazard ratio: 5.2; 95%; confidence interval: 2.0–13.3) (Klem et al., 2012).

Potentially, there is a wide range of possible antifibrotic treatment options in humans that could target the transforming growth factor-β1 (TGF-β1), endothelin-1 (ET-1), connective tissue growth factor (CTGF), ATII, and platelet-derived growth factor (PDGF) networks (Leask, 2010). Much of these interventions have been tested in animal models with various degrees of success. TGF-β1 is a well-recognized mediator of tissue healing and a major fibrosis-inducing cytokine that contributes to multiple fibroproliferative disorders, including cardiac fibrosis associated with heart failure. It is locally generated in most cell types, and several studies have shown beneficial value against the pathological development of fibrosis with suppression of TGF-β1 activity. In response to ATII, TGFβ1 expression is increased, converts fibroblasts into myofibroblasts, and generates extracellular matrix proteins such as type I collagen (Leask, 2007). For example, drugs that inhibit the renin–angiotensin–aldosterone system (RAAS) suppress cardiac fibrosis via improved hemodynamics and directly through inhibition of myofibroblast activity and collagen synthesis. Eplerenone, an ATII receptor blocker, greatly reduced cardiac fibrosis and arrhythmias inducibility in aged mice (Stein et al., 2010). In humans, RAAS activation increases ATII level, which is pro-arrhythmogenic, while RAAS inhibition reduces the arrhythmic risk, (Yusuf et al., 2000; Teo et al., 2004) likely by reduction of cardiac fibrosis as shown in animal studies. Recently, reduction of the glycoprotein endoglin, a co-receptor of TGFβ1 signaling in the cardiac fibroblast, correlates with an attenuation of cardiac fibrosis in patients with heart failure (Kapur et al., 2012). In these studies, an association between levels of endoglin, which are significantly increased in individuals with severe LV failure before implantation of the LV assist device (LVAD) but are dramatically reversed back to control levels in a separate cohort after LVAD placement. Because no direct assessment of the level of cardiac tissue fibrosis was done in this cohort, the clinical value of endoglin reduction in limiting the expansion of cardiac tissue fibrosis in humans is still to be determined (Benjamin, 2012). Similarly, block of mineralocorticoid receptors or endothelin receptors, (Mayyas et al., 2010) or block of TGF-beta synthesis with pirfenidone, (Lee et al., 2006; Nguyen et al., 2010) are shown to reduce LV fibrosis, dysfunction and arrhythmias after myocardial infarction in rats. These experimental findings are encouraging and suggest that reduction of cardiac fibrosis is not only possible, but also may reduce the risk of cardiac arrhythmias (Li et al., 2001; Vermes et al., 2003; Zeisberg et al., 2007; Ago et al., 2010; Leask, 2010; Massare et al., 2010; Tyralla et al., 2011).

## Conclusions

It is hoped that the basic research findings will be translated into clinical strategies to reduce cardiac arrhythmia risk in patients with heart disease. Antifibrotic therapy seems to hold great promise, as the independent role of fibrosis in the genesis of VT/VF is becoming increasingly recognized. Drugs directly targeting EAD formation may also be identified, now that the dynamical mechanisms involved have been clearer. For example, given the key role of I_Ca−L_ reactivation, drugs that shift the voltage dependence of steady-state activation and inactivation to reduce the amplitude of I_Ca−L_ window currents could potentially suppress EAD formation without adversely affecting normal excitation-contraction coupling. Similarly, specific blocker of the late INa current can also be useful in suppressing EADs as block of late Na channel openings does not interfere with excitation-contraction coupling. Finally, interventions designed to suppress oxidative stress or inhibit CaMKII activation in the setting chronic heart disease could also play a role.

### Conflict of interest statement

The authors declare that the research was conducted in the absence of any commercial or financial relationships that could be construed as a potential conflict of interest.

## Abbreviations

Na-Ca exchanger: sodium calcium exchanger
EADs: early afterdepolarizations
VT/VF: ventricular tachycardia and ventricular fibrillation respectively
PVT: polymorphic ventricular tachycardia
TdP: torsade de pointes
I_Ca-L_: L-type Ca current
PVC: premature ventricular complex
APD: action potential duration
ROS: reactive oxygen species
H_2_O_2_: hydrogen peroxide
CV: conduction velocity
ATII: Angiotensin II
NADPH: Nicotinamide adenine dinucleotide phosphate
Ca^2+^-CaMK: calcium calmodulin-dependent kinase
I_NaK_: electrogenic Na-K pump outward current
I_Na-L_: late sodium current
SERCA: sarcoplasmic endoplasium reticulum calcium uptake
ATPase: adenine trihosphatase
MsrA: methionine sulfoxide reductase A
Cx43: connexins43
SCD: sudden cardiac death
ICD: implantable cardioverter defibrillator
M-MF: myocyte myofibroblast coupling
Ito: transient outward current
ATX II: *Anemonia sulcata* toxin II
LV: left ventricle
DCM: dilated cardiomyopathy
HCM: hypertrophic cardiomyopathy
CVF: collagen volume fraction
PCP: procollagen type I carboxy-terminal proteinase
MRI: magnetic resonance imaging
TGF-β1: transforming growth factor-β1
ET-1: endothelin-1
CRGF: connective tissue growth factor
PDGF: platelet-derived growth factor
RAAS: renin–angiotensin–aldosterone system
LVAD: left ventricular assist device
ASK1: apoptosis signaling kinase 1
JNK: c-Jun N-terminal kinase
p38 kinase: p38 mitogen-activated kinase.
